# Endothelial Protection by Sodium-Glucose Cotransporter 2 Inhibitors: A Literature Review of In Vitro and In Vivo Studies

**DOI:** 10.3390/ijms25137274

**Published:** 2024-07-02

**Authors:** Nikolaos Mylonas, Panagiota Efstathia Nikolaou, Paschalis Karakasis, Panagiotis Stachteas, Nikolaos Fragakis, Ioanna Andreadou

**Affiliations:** 1 Laboratory of Pharmacology, Faculty of Pharmacy, National and Kapodistrian University of Athens, Panepistimioupolis, Zografou, 15771 Athens, Greece; mylonasn@pharm.uoa.gr (N.M.); nayanik@pharm.uoa.gr (P.E.N.); 2 Second Department of Cardiology, Aristotle University of Thessaloniki, Hippokration General Hospital of Thessaloniki, 54642 Thessaloniki, Greece; pakar15@hotmail.com (P.K.); staxteasp@gmail.com (P.S.); fragakis.nikos@googlemail.com (N.F.); 3 Outpatient Department of Cardiometabolic Medicine, Second Department of Cardiology, Aristotle University of Thessaloniki, 54642 Thessaloniki, Greece

**Keywords:** SGLT-2 inhibitors, endothelium, oxidative stress, nitric oxide, adhesion molecules, cytokines, inflammation, angiogenesis, endothelial dysfunction

## Abstract

Endothelial dysfunction often precedes the development of cardiovascular diseases, including heart failure. The cardioprotective benefits of sodium-glucose cotransporter 2 inhibitors (SGLT2is) could be explained by their favorable impact on the endothelium. In this review, we summarize the current knowledge on the direct in vitro effects of SGLT2is on endothelial cells, as well as the systematic observations in preclinical models. Four putative mechanisms are explored: oxidative stress, nitric oxide (NO)-mediated pathways, inflammation, and endothelial cell survival and proliferation. Both in vitro and in vivo studies suggest that SGLT2is share a class effect on attenuating reactive oxygen species (ROS) and on enhancing the NO bioavailability by increasing endothelial nitric oxide synthase activity and by reducing NO scavenging by ROS. Moreover, SGLT2is significantly suppress inflammation by preventing endothelial expression of adhesion receptors and pro-inflammatory chemokines in vivo, indicating another class effect for endothelial protection. However, in vitro studies have not consistently shown regulation of adhesion molecule expression by SGLT2is. While SGLT2is improve endothelial cell survival under cell death-inducing stimuli, their impact on angiogenesis remains uncertain. Further experimental studies are required to accurately determine the interplay among these mechanisms in various cardiovascular complications, including heart failure and acute myocardial infarction.

## 1. Introduction

Sodium-glucose cotransporter 2 inhibitors (SGLT2is) constitute a novel class of antidiabetics used against type 2 diabetes mellitus (T2DM). SGLT2 cotransporter belongs to the SLC5 family of active glucose transporters and is mainly expressed at the proximal tubule of the nephron. The physiological role of this transporter lies in the reabsorption of glucose and sodium, which is normally excreted in the urine during renal blood filtration. It is estimated that ~90% of the excreted glucose is reabsorbed from the urine back to the blood via SGLT2, while the remaining is being handled by SGLT1 [1]. Thus, inhibition of SGLT2 prevents glucose reabsorption, leading to blood glucose reduction [2]. Despite SGLT2is being initially developed as antidiabetics, they currently constitute the most recent addition to guideline-recommended medical therapy in heart failure (HF), irrespective of the spectrum of left ventricular ejection fraction (LVEF). The first signal indicating the cardiovascular benefits of SGLT2is was the assessment of cardiovascular morbidity and mortality in SGLT2is-treated patients with T2DM at high cardiovascular risk. The landmark clinical trial EMPA-REG OUTCOME [3] concluded that empagliflozin (EMPA) reduced HF hospitalization, cardiovascular death, and all-cause mortality in diabetic patients with high cardiovascular risk. The aforementioned findings exhibited consistency throughout subsequent clinical studies investigating the cardiovascular benefit of two other SGLT2is, namely canagliflozin (CANA) and dapagliflozin (DAPA) [4,5]. Further investigation of those drugs revealed a class effect on decreasing the risk of HF aggravation, irrespective of the presence of T2DM and the HF classification based on LVEF [6,7,8]. These findings led different medical associations, including the American College of Cardiology/American Heart Association Joint Committee and the Heart Failure Association of the European Society of Cardiology, to suggest the use of SGLT2is for the management of HF with reduced LVEF (HFrEF, Class I recommendation) and preserved LVEF (HFpEF, Class 2a recommendation) in their revised guidelines [9].

Considering the importance of the beneficial outcomes in heart failure, there is a growing interest in studying the specific pathways modified by SGLT2is, leading to cardiovascular protection. Towards this direction, several mechanisms have been suggested, including systemic and direct effects on the cardiorenal system. Natriuresis, diuresis, and blood pressure reduction are associated with improved ventricular loading conditions and decreased arterial stiffness, while glycosuria mediates weight loss. However, the extrarenal effects of SGLT2is are still obscure, and the mechanisms of enhanced myocardial bioenergetics, decreased myocardial fibrosis, and improved endothelial and microcirculatory function have not been fully elucidated yet. Nevertheless, systemic glucose unloading cannot be excluded as a contributor to the protective effects of this drug class. 

Coronary microvascular dysfunction, which is characterized by the inability of the microvasculature to augment coronary flow to meet the myocardial oxygen demand in the absence of epicardial coronary artery disease, is highly prevalent among the HFpEF population [10]. That is to say, it is observed in at least 75% of HFpEF patients and is associated with a higher incidence of cardiovascular death and HF hospitalizations [11]. EMPA has been found to alleviate endothelial and cardiac dysfunction in HFpEF patients by reducing inflammatory-oxidative pathways [12]. In addition, SGLT2is have been demonstrated to improve endothelial dysfunction in diabetic patients [13,14]. Despite clinical data systematically confirming the beneficial effect of SGLT2is on the endothelium, the evidence concerning the effect on microcirculation is conflicting. These results could be explained by the differences in the patient population, treatment protocols, the indexes assessed, as well as the absence of a thorough pre-protocol assessment of patients’ microcirculation status. An extensive analysis of the available clinical data regarding the protection induced by SGLT2is on the endothelium and microcirculation has been previously performed by Dimitriadis et al. [15]. That being said, comprehending the cardioprotective mechanisms of SGLT2is in relation to endothelial dysfunction will result in the development of more efficient approaches for managing microvascular dysfunction in HF and various other disorders that share a common pathophysiology with endothelial or microvascular disease, such as myocardial ischemia, diabetes, and chronic kidney disease [16,17,18]. The present literature review primarily focuses on the impact of SGLT2is on the endothelium and microcirculation by discussing the existing data obtained from in vitro studies conducted on endothelial cells, as well as in vivo studies in various animal models. The main objective is to expand our understanding of the following: (1) the molecular mechanisms of endothelial dysfunction, (2) the effect of SGLT2is on the endothelium based on in vitro and in vivo studies focusing on oxidative stress, inflammatory response, and angiogenesis, as well as (3) provide future perspectives on whether the effects of SGLT2is on the endothelium could explain the improvement in endothelial and cardiac dysfunction observed in clinical practice.

## 2. Molecular Mechanisms Contributing to Endothelial Dysfunction

The endothelium acts as a physical barrier between the vessel wall and lumen, playing a key role in vascular health. Under physiological conditions, the endothelium regulates vascular tone by synthesizing and releasing relaxing and contracting factors in balance while it actively regulates the extravasation of fluids, solutes, hormones, and macromolecules across its barrier, regulating blood fluidity. However, under various pathological conditions, such as hyperlipidemia, diabetes, and myocardial infarction, the endothelium regulates the inflammatory response. It serves as a supporting surface for the formation of the procoagulation complexes and the clotting to be achieved at the sites of vascular damage [19]. Endothelial dysfunction is identified as impaired vasodilation in response to endothelial stimuli such as acetylcholine or bradykinin and has been associated with cardiovascular risk factors such as T2DM, hypertension, and hyperlipidemia [20]. Microvascular dysfunction is provoked by both endothelium-dependent and independent processes, while experimental and clinical evidence underscores the pivotal role of the endothelium in regulating coronary vasoconstrictive reactivity and microvascular resistance [21]. The mechanisms that govern the development of endothelial dysfunction include the reduction in nitric oxide (NO) availability, excessive reactive oxygen species (ROS) production, and the production of vasoconstricting factors such as endothelin-1 [22,23,24,25,26].

NO supports endothelial cells’ viability, promotes vasorelaxation [27] and inhibits leukocyte and platelet recruitment and adhesion [28,29]. NO production in endothelial cells is primarily regulated by the phosphorylation of endothelial nitric oxide synthase (eNOS) enzyme via activation of the PI3K/Akt kinases. Subsequently, L-arginine is oxidized to L-citrulline and NO by eNOS [30]. Low levels of ROS contribute to maintaining endothelial cell proliferation and survival, as well as to modulating endothelial integrity and vascular function [31]. However, excessive ROS production uncouples eNOS, leading to reduced NO production and augmented superoxide anion (O_2_^−^)and peroxynitrite (ONOO^−^) formation in the endothelial cells [32]. 

Elevated ROS levels have been well-established to result in deleterious repercussions for the vasculature [33]. ROS promote vascular stiffness by increasing the levels of vasoactive molecules, such as matrix metalloproteinases (MMPs) and vascular endothelial growth factor (VEGF), thus inducing vascular remodeling and leading to blood pressure elevation [34]. The cascade of inflammation is also triggered by oxidative stress, which induces the expression of pro-inflammatory cytokines and chemokines, as well as the upregulation of intracellular adhesion molecule-1 (ICAM-1) and vascular cell adhesion molecule-1 (VCAM-1) from endothelial cells, leading to enhanced attraction and adherence of inflammatory cell to the endothelium [35]. Importantly, activation of the Src family of kinases (SFK) is also triggered, resulting in the phosphorylation of vascular endothelial-cadherin (VE-cadherin), which leads to its internalization, the breakdown of the adherent junctions, and increased endothelial permeability [36]. 

The pathomechanisms associated with cardiovascular risk factors, such as hyperglycemia and aging, intersect at the level of oxidative stress, leading to additional downstream dysregulation and damage of endothelial function. It is well documented that hyperglycemia induces protein and lipid glycation, leading to the formation of advanced glycation end products (AGEs). Upon formation, AGEs can interact with cell surface receptors for AGEs (RAGE), leading to increased ROS, tissue factor generation, and upregulation of VCAM-1 [37]. Moreover, excessive glucose concentration triggers metabolic pathways otherwise inactive or unsubstantial, such as the polyol pathway of glucose metabolism. This pathway involves the conversion of sorbitol to fructose, during which ROS is generated due to NADPH oxidation and depletion, as well as diminished glutathione.

Additionally, excessive ROS inhibits GAPDH activity, leading to an increase in the production of diacylglycerol, which in turn activates PKC and stimulates NADPH oxidases (NOXs) to create even more ROS [38]. Also, different stimuli, such as myocardial ischemia, increase the number and function of sodium–hydrogen exchangers (NHE) in endothelial cells, which facilitates the entry of Na^+^ ions and triggers the elevation of intracellular Ca^2+^ concentrations via activating sodium–calcium exchangers (NCX) [39]. The elevation in cytosolic Ca^2+^ subsequently triggers the activation of the PKC-NOXs pathway, further promoting ROS generation [40]. 

Finally, apart from nitro-oxidative stress accumulation and mitochondrial damage, advanced age is associated with the senescence process of endothelial cells. Senescence leads to increased expression of various pro-inflammatory cytokines, including tumor necrosis a (TNF-α). The molecular cascades mentioned above compromise vascular tone; thus, the current hypothesis points to their crucial role in the pathogenesis of HF [41].

In summary, the primary mechanisms that promote the development of endothelial dysfunction include the reduction in NO bioavailability, the increase in ROS formation, and the inflammatory response, which lead to endothelial cell senescence and death. In the next section of this review, we discuss how SGLT2is affect the aforementioned molecular mechanisms to alleviate endothelial dysfunction and provide cardiovascular benefit. 

## 3. Suggested Mechanisms for SGLT2is Endothelial Protection

A large number of in vitro and in vivo studies have been conducted to evaluate the effect of SGLT2is on the endothelium, and the main findings of those studies are recapitulated in Table 1 and Table 2. Those studies propose a variety of mechanisms by which SGLT2is attenuate microvascular and endothelial dysfunction. These mechanisms, extensively analyzed below, mainly include antioxidant effects, improved NO production, and anti-inflammatory and angiogenetic effects.

### 3.1. Anti-Oxidative Effect of SGLT2is

#### 3.1.1. In Vitro Studies

Several in vitro studies have been performed in an effort to elucidate the anti-oxidative effect of SGLT2is. EMPA prevented hyperglycemia-induced mitochondrial disruption and preserved endothelial barrier function in murine cardiac microvascular endothelial cells (CMECs) through inhibition of mitochondrial fission [42]. The ability of SGLT2is to preserve the endothelial cell barrier has also been shown in another study conducted in human coronary artery endothelial cells (HCAECs) subjected to enhanced cyclic stretch [43]. In this study, EMPA, DAPA, and CANA preincubation prevented VE-cadherin depletion and suppressed stretch-induced cell permeability via a reduction in mechanical force-induced oxidative damage. In the same study, EMPA co-administration with cariporide or GKT136901, a sodium–hydrogen exchanger 1 (NHE1) inhibitor, and an NADPH oxidases (NOXs) inhibitor, respectively, suggested that the anti-oxidative effect of SGLT2is can be attributed to both NHE1 and NOX inhibition [43].

The notion that NHE mediates, at least in part, the anti-oxidative properties of SGLT2is has been strongly supported by a number of studies in various cellular models. Direct inhibition of the NHE by SGLT2is was first observed in cardiomyocytes cultured under both hyperglycemic and normoglycemic conditions [42,43]. More recently, Cappetta et al. [44] provided proof of NHE inhibition in “non-stimulated” human umbilical vein endothelial cells (HUVECs) treated with DAPA, while Uthman et al. [45] proved that EMPA lowers ROS production by suppressing NHE activity in human endothelial cells activated by TNF-α. On the contrary, Juni et al. [46] exposed human (CMECs) to uremic serum collected from patients with chronic kidney disease and observed a stronger ROS-suppressing capacity by EMPA (1 μM) than with cariporide (10 μM) treatment (63% vs. 38%), implying that part of the anti-oxidative effect of EMPA is not linked to NHE inhibition. Borrielo et al. recently provided an overview of the possible effects of SGTL2is on NHE, indicating that mitochondrial and sodium dynamics alterations may account for the protective effects of SGLT2is in various cell types, including endothelial cells [47].

#### 3.1.2. In Vivo Studies

Preclinical studies involving various animal models of cardiac disease have provided further evidence regarding the anti-oxidative effect of SGLT2is. One of the first preclinical in vivo studies [42] provided evidence that EMPA attenuates ROS formation (both cytosolic and mitochondrial) in an adenosine monophosphate (AMP)-activated protein kinase (AMPK)-dependent manner. Additionally, in a streptozotocin (STZ)-induced diabetic rat model treated with EMPA (10 or 30 mg/kg/day orally for 7 weeks), EMPA suppressed oxidative stress and prevented the development of endothelial dysfunction in the aortic vessels [48]. Since then, other groups have confirmed the SGLT2is-induced suppression of nitro-oxidative stress in the arteries of other diabetic animal models, such as the db/db mice and the Zucker diabetic fatty (ZDF) rats [49,50]. Mechanistically, this protective effect induced by EMPA treatment has been attributed to the suppression of NOX2 and NOX4 mRNA levels in the aortic and renal endothelium, respectively. Correspondingly, investigation of the anti-oxidative properties of other members of the SGLT2is class, such as CANA and DAPA, in diabetic animals, has led to the same conclusions, rendering ROS suppression as a class effect of the SGLT2is [51,52,53]. However, since all of the studies mentioned above were conducted in diabetic animals and pharmacological SGLT2 inhibition was accompanied by a decrease in their blood glucose levels, it might be assumed that the observed ROS inhibitory effect is mediated by glucose unloading.

Recently, we proved that chronic EMPA pretreatment (10 mg/kg/day orally for 6 weeks) reduces infarct size and suppresses oxidative stress biomarkers MDA and protein carbonyls in myocardial tissue obtained from nondiabetic mice subjected to acute myocardial infarction. In the same study, EMPA increased superoxide dismutase 2 levels, and this was attributed to increased phosphorylation and activation of STAT3 at Tyr705, which modulates many anti-oxidative and anti-apoptotic genes [54].

Finally, a more recent study [55] revealed that a 7-day pretreatment with EMPA reduced infarct size and improved cardiac function by suppressing oxidative stress and microvascular obstruction in a porcine model of acute myocardial infarction. Furthermore, perivascular intravenous infusion of the ketone body beta-hydroxybutyrate provoked similar results, implying the implication of the heart’s metabolic shift towards ketone body consumption in SGLT2is-mediated alleviation of oxidative stress. Importantly, in this study, beta-hydroxybutyrate levels were not measured in plasma or myocardial biopsies collected from EMPA-treated animals. Thus, ketonemia and alteration of the metabolic substrate towards ketone bodies can only be assumed based on previously published studies [56,57].

In summary, the great majority of data obtained from in vitro and in vivo studies recognize suppression of oxidative stress as a pivotal contributor to SGLT2is-induced endothelial protection. However, a discrepancy exists regarding the proposed molecular mechanisms governing this effect.

### 3.2. Improved NO Production and Vasodilation

Under various pathological conditions, such as myocardial ischemia, heart failure, and diabetes, endothelial NO concentration is suppressed, primarily due to eNOS dephosphorylation, ROS-induced eNOS uncoupling, and increased NO consumption [58,59]. Moreover, cardiac dysfunction induced by permanent left coronary artery ligation was alleviated in transgenic mice overexpressing eNOS in their vascular endothelium, further highlighting the significance of eNOS and NO production for cardiovascular health [57,60]. Hence, a series of in vitro and in vivo studies conducted on SGLT2is have focused their attention on NO production and function.

#### 3.2.1. In Vitro Studies

EMPA and DAPA treatment (1 μM) inhibited the suppressed NO levels in human CMECs exposed to TNF-α or uremic serum derived from patients with chronic kidney disease [61]. In the same studies, authors observed a reduction in oxidative stress in EMPA and DAPA-treated CMECs, but phosphorylated eNOS levels remained unchanged, thus proposing that increased NO bioavailability is mediated by the SGLT2is’ ROS inhibitory capacity rather than by eNOS activation. Contradictory results have been provided by other studies conducted in HCAECs subjected to hypoxia/reoxygenation. Specifically, EMPA and DAPA pretreatment (10 μM for 12 h–24 h) significantly improved cell viability and function by increasing eNOS phosphorylation and activity [62]. DAPA was also evaluated on HUVEC exposed to H_2_O_2_ and restored eNOS serine phosphorylation and sirtuin 1 (SIRT1) expression leading to decreased endothelial senescence-associated markers β-galactosidase (SA-β-gal), p21, and p53. In parallel, DAPA reduced eNOS phosphorylation through SIRT1 up-regulation, indicating that SIRT1 could be involved in enhancing eNOS activity by SGLT2is [63]. Lastly, in another study conducted in isolated rat coronary arteries, dapagliflozin induced vasorelaxation, but this effect was independent of SGLT2 inhibition, eNOS activation, and K^+^ channels [64]. Notably, the discrepancy observed in eNOS activation can be explained by the different stressors (TNF-α and uremic serum vs. hypoxia/reoxygenation) used among those studies.

#### 3.2.2. In Vivo Studies

In vivo studies have confirmed the increased NO bioavailability and vasodilation induced by SGLT2is, but the effect on eNOS signaling is again contradictory. It has been reported that EMPA improves diastolic cardiac function via enhancement of NO bioavailability and eNOS activity in a swine model of HF induced by myocardial ischemia/reperfusion injury [65]. Accordingly, similar results have been obtained on eNOS activation and NO bioavailability in diabetic mice and metabolic syndrome ZSF1 rats treated with DAPA (60 mg/kg/day orally for 8 weeks) or EMPA (30 mg/kg/day orally for 6 weeks), respectively [66,67]. Importantly, in both studies, functional analysis of the coronary and the mesenteric arteries revealed improved relaxation and function in animals treated with EMPA or DAPA. Accordingly, DAPA chronic treatment in db/db mice enhanced endothelium-dependent vasorelaxation and increased plasma NO concentration, suggesting that DAPA ameliorates endothelial dysfunction by enhancing NO bioavailability. In contrast, EMPA administration in diabetic mice led to increased eNOS activation in their CMECs [63]. 

Taken together, the majority of in vitro and in vivo studies confirm that vasodilation represents a significant contributor to SGLT2is-induced endothelial protection. However, a discrepancy is observed among those studies regarding the role of activation of eNOS and NO bioavailability in this protection.

### 3.3. Anti-Inflammatory Effect of SGLT2is

SGLT2is have been well documented to exhibit anti-inflammatory properties in various types of cardiac cells, such as cardiomyocytes, cardiac fibroblasts, immune cells, endothelial, and smooth muscle cells [68]. Importantly, SGLT2is restore endothelial dysfunction by attenuating inflammation in various in vitro and in vivo models of disease with mechanisms that either overlap or are independent of oxidative stress and NO availability.

#### 3.3.1. In Vitro Studies

One of the first in vitro studies to investigate the anti-inflammatory effect of SGLT2is was conducted by Gaspari et al. [69] and showed that low-dose DAPA treatment (1.0–5.0 nM) attenuated the increase in ICAM-1 and VCAM-1 adhesion molecules in HUVECs exposed to hyperglycemia or TNF-α for 24 h. Accordingly, EMPA (50 μM for 24 h) prevented the TNF-α-induced adhesion of NB4 leukocytes onto cultured human abdominal aortic endothelial cells (HAAECs), further demonstrating the protective role of SGLT2is on endothelial barrier integrity and function without altering ICAM-1 and VCAM-1 expression [70]. However, several other groups have reported a neutral effect of EMPA (1 μM) and DAPA (1 μM) on the TNF-α-induced expression of adhesion molecules ICAM-1 and VCAM-1 in various in vitro endothelial cell models [61,71]. The discrepancy observed in VCAM-1 and ICAM-1 expression levels can be explained by differences in the concentration of SGLT2is used (1.0–5.0 nM vs. 1 μM, respectively), as well as the duration of TNF-α treatment (24 h vs. 4 h).

An inhibitory effect on IL-6 secretion has been observed in HCAECs pretreated with CANA (3 μM or 10 μM for 16 h) and then subjected to lipopolysaccharide (LPS, 1 μg/mL for 3 h) treatment [72]. Surprisingly, in the same study, EMPA and DAPA had no effect on IL-6 release, and this CANA-specific effect coincided with its ability to induce AMPK phosphorylation and activation. This unique, additional anti-inflammatory effect of CANA has also been described by another group that provided proof that this effect is mediated by SGLT1 inhibition since CANA is one of the least selective SGLT2is and, thus, has a high potency for inhibiting SGLT1 in clinically relevant doses [73]. In contrast, one study [74] reported that DAPA suppressed IL-6 and IL-8 secretion in LPS-treated (20 ng/mL for 24 h) HUVECs via NF-κB and toll-like receptor 4 suppression, and this divergent result could be attributed to LPS exposure protocol (1 μg/mL for 3 h vs. 20 ng/mL for 24).

#### 3.3.2. In Vivo Studies 

Despite the neutral or inconclusive results from in vitro studies, SGLT2is have been shown to prevent the upregulation of several adhesion molecules, such as ICAM-1, VCAM-1, E-selectin, and P-selectin in various in vivo models of cardiac disease [50,62,75,76], indicating a class effect for this mechanism of endothelium protection. Additionally, several in vivo studies have reported a reduction in circulating inflammatory markers induced by SGLT2is. Treatment of db/db diabetic mice with DAPA (60 mg/kg/day orally for 8 weeks) significantly suppressed several inflammatory markers (CCL-2, CCL-5, IL-1β, IL-6, and IL-17). In normoglycemic mice on a high-salt diet, DAPA (0.1 mg/kg/day for 6 weeks) prevented NF-κB, CCL2, IL-6, and E-selectin upregulation [77]. Moreover, EMPA administration (10 and 30 mg/kg/day for 6 weeks) suppressed the expression of many inflammatory markers, such as cyclooxygenase-2 (COX-2), inducible NOS (iNOS), and interferon-γ (IFN-γ) and improved endothelial function in the thoracic aorta in Zucker diabetic rats [50]. Lastly, CANA administration (10 mg/kg/day orally for 5 weeks) in ApoE^−/−^ mice fed with a high-fat diet led to a significant reduction in aortic plaque formation, as well as a suppression of CCL-2 mRNA levels in their aortic lesions [77].

To sum up, various in vitro and in vivo disease models propose alleviation of the inflammatory response as a significant contributor to SGLT2is-induced protection against endothelial dysfunction. Yet, the exact molecular mechanisms that govern the anti-inflammatory properties of SGLT2ist are still a matter of debate, and more studies are needed to elucidate the exact molecular mechanisms for this effect [78].

### 3.4. Impact of SGLT2is on Endothelial Cell Survival and Angiogenesis

The impact of SGLT2is on endothelial cell viability upon various stressors and angiogenesis has been the center of investigation for both in vitro and in vivo studies. 

#### 3.4.1. In Vitro Studies

An in vitro study conducted by our group revealed that EMPA treatment in HMECs subjected to hypoxia/reoxygenation increased cell viability, with the protective effect being STAT-3 dependent [79]. In accordance with those findings, DAPA treatment (0.01–100 μM) preserved cell viability in a dose-dependent manner in HCAECs exposed to hypoxia/reoxygenation. Importantly, VEGF levels were found to be suppressed in HCAECs exposed to hypoxia/reoxygenation, but this effect was altered by DAPA treatment [62]. On the contrary, CANA treatment (10–50 μM) inhibited proliferation and migration in HUVECs and human aortic endothelial cells (HAECs) cultured under no-stress conditions, and the same effect was observed upon EMPA or DAPA treatment (30–50 μM) [80]. These opposing effects of CANA on angiogenesis might be explained by several differences in the experimental conditions used among those studies. For example, EMPA and DAPA presented anti-apoptotic and pro-angiogenic effects when endothelial cells were cultured under “stress” conditions, such as hypoxia/reoxygenation, which blunts cell viability and proliferative capacity. In contrast, the anti-proliferative effect of CANA has been reported in “non-stimulated” cells. Moreover, the anti-proliferative effect of EMPA and DAPA was observed only when high concentrations were used (30–50 μM), which are not clinically relevant. Finally, respective selectivity for SGLT2 over SGLT1 should not be omitted since SGLT1 is highly expressed in ECs, and CANA presents a significant SGLT1 inhibitory effect, which might interfere with their glucose uptake capacity [81].

#### 3.4.2. In Vivo Studies

One of the first in vivo studies to examine the role of SGLT2is on angiogenesis was conducted by Zhou et al. [42] and demonstrated that EMPA administration (10 mg/kg/day for 20 weeks) in STZ-treated diabetic mice promoted CMECs migration and neovascularization thus alleviating diabetes-induced cardiac microvascular dysfunction and improving cardiac perfusion. Mechanistically, this effect was attributed to the suppression of F-actin depolymerization, F-actin stabilization, and inhibition of mitochondrial fission. However, opposing data were obtained from a study conducted by Soares et al. [82], which reported that EMPA administration (14 mg/kg/day for 6 weeks) improved endothelial function and reduced arterial stiffness via decreasing F-actin and P-cofilin levels in the mesenteric arteries of an aging murine model.

Conclusively, the effect of SGLT2is on endothelial cell proliferation and migration varies depending on the different tissues and disease models used. Thus, more studies are needed to interpret and explain the observed differences.

**Table 1:** In vitro studies regarding the direct effect of SGLT2 inhibitors on endothelial function. ↓ indicates decrease, while ↑ indicates increase. AMPK, AMP-activated protein kinase; CANA, canagliflozin; CMECs, cardiac microvascular endothelial cells; DAPA, dapagliflozin; EMPA, empagliflozin; eNOS, endothelial nitric oxide synthase; β-gal, β-galactosidase; HCAECs, human coronary artery endothelial cells; HUVECS, human umbilical vein endothelial cells; HMECs, human microvascular endothelial cells; HAECs, human aortic endothelial cells; ICAM-1, intracellular adhesion molecule-1; IL, interleukins; NHE, sodium–hydrogen exchanger; NO, nitric oxide; NF-κB, nuclear factor kappa-light-chain-enhancer of activated B cells; NOX, nicotinamide adenine dinucleotide phosphate oxidase-4; ROS, reactive oxygen species; STAT3, signal transducer and activator of transcription 3; SIRT-1, sirtuin-1; TNF-α, tumor necrosis factor-α; VCAM-1, vascular cell adhesion molecule-1; VE-cadherin, vascular endothelial-cadherin; VEGF, vascular endothelial growth factor.

Drug (Concentration)	Experimental Model	Stimulant	Major Findings	Ref.
EMPA (1 μM)/6 h	HCAECs HUVECs	TNF-α (10 ng/mL)/6 h	↓ NHE activity ↓ ROS	[45]
EMPA (1 μM)/6 h	CMECs	Uraemic serum (15%)/6 h	↓ ROS ↑ NO bioavailability	[71]
EMPA (1 μM)/6 h	CMECs	TNF-α (10 ng/mL)/6 h	↓ ROS ↑ NO	[61]
EMPA (10 μM)/12 h	HCAECs	Hypoxia/Reoxygenation	↓ ROS ↓ Mitochondrial fission ↓ ICAM-1 ↑ VE-cadherin ↑ p-eNOS	[83]
EMPA (50 μM)/24 h	HAECs	TNF-α (10 ng/mL)/24 h	↓ Leukocyte–endothelium adhesion	[70]
EMPA (500 nM)/24 h	HMECs	Hypoxia/Reoxygenation	↓ ROS ↑ p-STAT3 ↑ Cell viability	[79]
DAPA (1 μM)/24 h	HUVECs	-	↓ NHE activity	[44]
DAPA (10 μM)/12 h	HCAECs	Hypoxia/Reoxygenation	↓ Mitochondrial fission ↓ ICAM-1 ↑ p-eNOS ↑ VEGF ↑ Cell Survival	[62]
DAPA (10 μM)/72 h	HUVECS	H_2_O_2_ (100 µM)/1 h	↓ β-gal, p21, p53 ↓ Senescence ↑ SIRT-1 ↑ p-eNOS	[63]
DAPA (1–5 nM)/24 h	HUVECs	TNF-α (10 ng/mL)/24 h	↓ ICAM-1 ↓ VCAM-1	[69]
DAPA (0.05–0.5 μM)/24 h	HUVECs	LPS (20 ng/mL)/24 h	↓ IL-6, IL-8 ↓ NF-κB	[74]
CANA (3 or 10 μM)/16 h	HCAECs	LPS (1 μg/mL)/3 h	↓ IL-6 ↑ p-AMPK	[72]
EMPA (1 μM)/ 24 h DAPA (1 μM)/ 24 h CANA (3 μM)/ 24 h	HCAECs	Cyclic stretch (1 Hz, 10%)/24 h	↓ NHE and NOX activity ↓ ROS ↓ Cell permeability ↑ VE-cadherin	[43]
EMPA (30–50 μM)/ 1–3 days DAPA (30–50 μM)/ 1–3 days CANA (10–50 μM)/1–3 days	HUVECs HAECs	-	↓ Angiogenesis ↓ Cell viability	[80]

**Table 2:** In vivo studies regarding the direct effect of SGLT2 inhibitors on endothelial function. ↓ indicates decrease, while ↑ indicates increase. CANA, canagliflozin; CCL-2, chemokine (C-C motif) ligand-2; COX-2, cyclooxygenase-2; DAPA, dapagliflozin; EMPA, empagliflozin; eNOS, endothelial nitric oxide synthase; ICAM-1, intracellular adhesion molecule-1; IL, interleukins; iNOS, inducible nitric oxide synthase; MDA, malondialdehyde; NO, nitric oxide; NF-κB, nuclear factor kappa light chain enhancer of activated B cells; NOX, nicotinamide adenine dinucleotide phosphate oxidase-4; ROS, reactive oxygen species; STAT3, signal transducer and activator of transcription 3; STZ, streptozotocin; SOD-2, superoxide dismutase-2; VCAM-1, vascular cell adhesion molecule-1.

Drug (Dosage)	Experimental Model	Stimulant/Intervention	Major Findings	Ref.
EMPA (10 mg/kg/day)/20 weeks	C57BL/6J mice (Diabetic)	STZ (50 mg/kg/day)/5 days	↓ ROS ↓ Mitochondrial fission and fusion ↓ Senescence ↑ Angiogenesis	[42]
EMPA (10 or 30 mg/kg/day)/ 7 weeks	Wistar rats (Diabetic)	STZ (60 mg/kg)	↓ ROS, NOX-1, NOX-2 ↓ IL-6, CCL-2 ↓ ICAM-1 ↑ p-eNOS	[48]
EMPA (10 or 30 mg/kg/day)/ 6 weeks	ZDF rats (Diabetic)	-	↓ ROS ↓ ICAM-1 ↓ COX-2, iNOS	[50]
EMPA (10 mg/kg/day)/ 6 weeks	C57BL/6J mice (Nondiabetic)	Myocardial ischemia/reperfusion	↓ Infarct size ↓ MDA ↓ Protein carbonyls ↑ p-STAT3 ↑ SOD-2	[79]
EMPA (10 mg/day)/ 7 days	Yorkshire pigs (Nondiabetic)	Myocardial ischemia/reperfusion	↓ Infarct size ↓ ROS ↑ Cardiac function	[55]
EMPA (14 mg/kg/day)/ 6 weeks	C57BL/6J Aged mice (Nondiabetic)	-	↓ ROS, MDA ↓ Arterial stiffness ↑ p-eNOS	[82]
EMPA (10 mg/day)/ 2 months	Yorkshire pigs (Nondiabetic)	Myocardial ischemia/reperfusion	↑ Cardiac function ↑ NO ↑ p-eNOS	[65]
EMPA (30 mg/kg/day)/ 6 weeks	ZSF1 rats (Diabetic)	-	↑ NO ↑ p-eNOS	[67]
DAPA (60 mg/kg/day)/ 8 weeks	db/db mice (Diabetic)	-	↓ IL-1β, IL-6, CCL-2 ↑ p-eNOS	[66]
DAPA (1 mg/kg/day)/ 8 weeks	db/db mice (Diabetic)	-	↑ NO ↑ p-eNOS	[63]
DAPA (0.1 mg/kg/day)/ 6 weeks	Dahl salt-sensitive rats (Nondiabetic)	8% NaCl special diet	↓ NF-κB, IL-6, CCL-2, E-selectin	[66]
CANA (20 mg/kg/day)/ 6 weeks	C57BL/6J mice (Diabetic)	STZ (150 mg/kg, single dose)	↓ ROS ↓ MDA	[53]
CANA (10 mg/kg/day)/ 5 weeks	ApoE^−/−^ mice (Diabetic)	High-fat diet	↓ Atherosclerotic plaques ↓ CCL-2 ↓ VCAM-1	[77]

## 4. Summary and Future Perspectives

The starting point of the present review was based on the realization of the outstanding cardioprotective effects of SGLT2is in patients with established heart failure or with high cardiovascular risk, irrespective of the type of HF or the presence of diabetes, which is a class effect of this drug class [3,4,7,8,84]. In addition, an accumulating body of evidence suggests that endothelial dysfunction is the cornerstone of microvascular dysfunction, consequently culminating in cardiovascular manifestations, such as HF [85]. Endothelium-dependent vasodilation is reduced in patients with both HFrEF and HFpEF [86]. Moreover, factors that disrupt the balance between NO and superoxide production in the vascular bed are likely to contribute to the pathophysiology of HFrEF and HFpEF, reviewed elsewhere [85]. Therefore, in this review, we summarized the preclinical studies pointing to the endothelial-mediated protection by SGLT2is, bridging the realms of clinical observations and experimental research.

To our knowledge, SGLT2 is not expressed in human ECs nor in the human heart under normal conditions [87,88]. However, SGLT2 expression levels have been found to be significantly upregulated in isolated porcine coronary artery endothelial cells exposed to high glucose or angiotensin II [89,90]. In accordance with those studies, cardiac SGLT2 has been found to be transiently expressed in a murine model of myocardial infarction, and this effect was obvious only in the ischemic area, suggesting a correlation between SGLT2 and myocardial damage [91]. More recently, SGLT2 was identified in the left ventricular tissue of cardiac patients subjected to valve surgery, and its levels were positively correlated with markers of cardiac remodeling, inflammation, and oxidative stress [92,93].

Conversely, the results obtained from studies with SGLT2 knockout animals are few but indicate that the cardioprotective mechanisms can also be SGLT2 independent. Our group has shown that in mice subjected to myocardial ischemia/reperfusion, SGLT2 global knockout does not confer cardioprotection, while EMPA pretreatment (10 mg/kg/day for 7 days) suppressed infarct size to the same extent in SGLT2-KO and wild-type mice [94]. Accordingly, total body SGLT2 deficiency itself does not protect against cardiac dysfunction development in two separate HF models induced by myocardial infarction and transverse aortic constriction [95]. Another study by our group indicated that 7 days of pretreatment with EMPA, DAPA, and ertugliflozin at stoichiometrically equivalent doses led to SGLT2 inhibition to a similar extent. Yet, only EMPA and DAPA conferred cardioprotection in terms of infarct size reduction. These data may also indicate drug-specific effects that could be related to cardioprotection. Hence, the aforementioned studies suggest that cardioprotection induced by SGLT2is may be independent of the SGLT2, and other molecular mechanisms, which have not yet been fully elucidated, are responsible for this effect [54]. However, the extrarenal effects of these drugs are still obscure, and further studies are needed to clarify if these drugs have other potential targets in vitro and in vivo with sufficient reliability.

**Figure 1:**
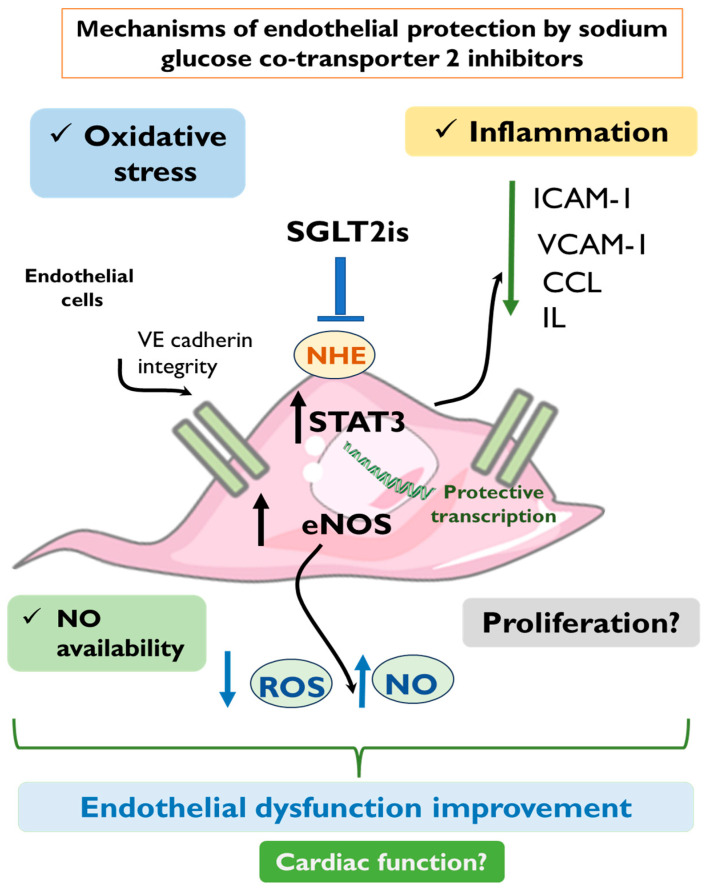
Proposed mechanisms of endothelial protection by sodium-glucose cotransporter 2 inhibitors. SGLT2is have been proposed to regulate oxidative stress, inflammatory response, NO bioavailability, and proliferation of endothelial cells. The molecular mechanisms affected by SGLT2is include STAT3 activation, eNOS phosphorylation, preservation of VE-cadherin integrity, as well as suppression of ICAM-1, VCAM-1, CCL, and IL in the endothelium. CCL, chemokine (C-C motif) ligand; eNOS, endothelial nitric oxide synthase; ICAM-1, intracellular adhesion molecule-1; IL, interleukins; NHE, sodium–hydrogen exchanger; NO, nitric oxide; ROS, reactive oxygen species; SGLT2is, sodium–glucose cotransporter 2 inhibitors; STAT3, signal transducer and activator of transcription 3; VCAM-1, vascular cell adhesion molecule-1.

Therefore, despite SGLT2is’ cardioprotection being well documented in clinical practice, extensive research investigation has been performed regarding the underlying molecular mechanisms of cardioprotection, as well as the identification of the primary cellular targets responsible for mediating SGLT2is’ cardioprotective effects. While most of the previously published preclinical studies focus on the direct effect of SGLT2is on cardiomyocytes [96,97,98,99,100], more and more evidence is accumulating regarding the contribution of non-cardiomyocyte populations for SGLT2is-induced cardioprotection. That is to say, SGLT2is have been shown to directly affect cardiac fibroblasts [101,102,103], macrophages [104,105,106], and smooth muscle cells [107,108,109]. Importantly, a large proportion of studies emphasize endothelial cells as a promising therapeutic target directly affected by SGLT2is. The favorable effect of SGLT2is against endothelial dysfunction is supported by both in vitro and in vivo preclinical studies. Our review indicates that the mechanisms of SGLT2is-induced endothelial protection involve direct effects on endothelial cells, leading to alleviation of oxidative stress, enhanced NO availability, and increased endothelial cell survival. In parallel, systemic in vivo models of HF or myocardial injury indicate a consistent attenuation of adhesion molecules and chemokine release, indicating SGLT2is’ protective effects against inflammation. Despite there being a great pool of preclinical studies supporting the protective role of SGLT2is on the endothelium, the exact molecular targets directly affected by SGLT2is are not reliably recognized. This limitation aligns with the fact that the SGLT2 transporter’s expression in the blood vessels or the endothelial cells has yet to be demonstrated by direct means. The proposed mechanisms of SGLT2is-induced endothelial protection are summarized in Figure 1. 

More recently, another groundbreaking clinical trial was published, providing further evidence regarding the cardiovascular benefit that EMPA possesses. Specifically, this study was performed in patients hospitalized for acute myocardial infarction who were at risk of HF development, and EMPA (10 mg/day) was administered within 14 days of admission [110]. Despite this study concluding that the composite outcome of heart failure hospitalization or death from any cause was not influenced by EMPA administration, a recent meta-analysis examining the individual components of the composite primary endpoint found that EMPA reduced both first and total heart failure hospitalizations [111]. Taken together, considering the high prevalence of coronary microvascular dysfunction among patients with cardiac disease [10,112], more well-designed studies are still required to elucidate the full spectrum of SGLT2is-induced cardiovascular benefits in the endothelium.

## Data Availability

No new data were created or analyzed in this study. Data sharing is not applicable to this article.

## Abbreviations

AGEs	Advanced glycation end products
CANA	Canagliflozin
CCL	Chemokine (C-C motif) ligand
CMECs	Cardiac microvascular endothelial cells
DAPA	Dapagliflozin
COX-2	Cyclooxygenase-2
EMPA	Empagliflozin
eNOS	Endothelial nitric oxide synthase
HAECs	Human aortic endothelial cells
HCAECs	Human coronary artery endothelial cells
HMECs,	Human microvascular endothelial cells
HUVECs	Human umbilical vein endothelial cells
HF	Heart failure
HFpEF	Heart failure with preserved ejection fraction
HFrEF	Heart failure with reduced ejection fraction
ICAM-1	Intracellular adhesion molecule-1
IFN-γ	Interferon-γ
IL	Interleukins
iNOS	Inducible nitric oxide synthase
LVEF	Left ventricular ejection fraction
NHE1	Sodium–hydrogen exchanger 1
NCX	Sodium–calcium exchangers
NO	Nitric oxide
NOXs	Nicotinamide adenine dinucleotide phosphate oxidases
RAGE	Receptors for advanced glycation end products
ROS	Reactive oxygen species
SGLT2is	Sodium-glucose cotransporter 2 inhibitors
SIRT1	Sirtuin 1
SFK	Src family of kinases
STAT3	Signal transducer and activator of transcription 3
T2DM	Type 2 diabetes mellitus
VCAM-1	Vascular cell adhesion molecule-1
VEGF	Vascular endothelial growth factor
VE-cadherin	Vascular endothelial-cadherin
